# Birth Anomalies in Monozygotic and Dizygotic Twins: Results From the California Twin Registry

**DOI:** 10.2188/jea.JE20170159

**Published:** 2019-01-05

**Authors:** Yang Yu, Wendy Cozen, Amie E. Hwang, Myles G. Cockburn, John Zadnick, Ann S. Hamilton, Thomas Mack, Jane C. Figueiredo

**Affiliations:** 1 Department of Preventive Medicine, Keck School of Medicine, University of Southern California, Los Angeles, CA, USA; 2 Norris Comprehensive Cancer Center, Keck School of Medicine, University of Southern California, Los Angeles, CA, USA; 3 Department of Epidemiology, Colorado School of Public Health and AMC/CancerCure Chair, Cancer Prevention and Control, University of Colorado Cancer Center, Aurora, CO, USA; 4 Department of Medicine, Samuel Oschin Comprehensive Cancer Institute, Cedars-Sinai Medical Center, Los Angeles, CA, USA

**Keywords:** birth anomaly, twin, pairwise concordance, environment, smoking

## Abstract

**Background:**

Inherited factors and maternal behaviors are thought to play an important role in the etiology of several congenital malformations. Twin studies can offer additional evidence regarding the contribution of genetic and lifestyle factors to common birth anomalies, but few large-scale studies have been reported.

**Methods:**

We included data from twins (20,803 pairs) from the population-based California Twin Program. We compared concordance in monozygotic (MZ) to dizygotic (DZ) twins for the following birth anomalies: clubfoot, oral cleft, spina bifida, muscular dystrophy, deafness, cerebral palsy, strabismus, and congenital heart defects. Each birth anomaly was also examined for the associations with birth characteristics (birthweight and birth order) and parental exposures (age, smoking, and parental education).

**Results:**

The overall prevalence of any selected birth anomaly in California twins was 38 per 1,000 persons, with a slightly decreasing trend from 1957–1982. For pairwise concordance in 6,752 MZ and 7,326 like-sex DZ twin pairs, high MZ:DZ concordance ratios were observed for clubfoot (CR 5.91; *P* = 0.043) and strabismus (CR 2.52; *P* = 0.001). Among the total 20,803 pairs, parental smoking was significantly associated with risk of spina bifida (OR 3.48; 95% CI, 1.48–8.18) and strabismus (OR 1.61; 95% CI, 1.28–2.03). A significant quadratic trend of increasing risk for clubfoot, spina bifida, and strabismus was found when examining whether father smoked, mother smoked, or both parents smoked relative to non-smoking parents (*P* = 0.029, 0.026, and 0.0005, respectively).

**Conclusions:**

Our results provide evidence for a multifactorial etiology underlying selected birth anomalies. Further research is needed to understand the biological mechanisms.

## INTRODUCTION

Approximately 6% of total births worldwide and 3% of newborns in the United States are born with a structural or genetic birth anomaly annually.^1^ The most common birth anomalies include congenital heart defects (86.4 per 10,000), oral cleft (14 per 10,000), and clubfoot (10 per 10,000).^2^^–^^4^ Although birth anomalies have distinct etiologies, they nevertheless share some common underlying risk factors, including genetic and parental lifestyle risk factors. Advanced maternal age is associated with an increasing prevalence of birth anomalies (ie, Down syndrome),^5^^–^^7^ and maternal smoking is linked to an increased risk of oral cleft, clubfoot, and congenital heart defects.^8^ Aside from age and maternal smoking, other risk factors, such as other maternal conditions, paternal smoking, parental education, or birth characteristics, have been linked to certain birth anomalies. However, those factors have seldom been tested for a wide breadth of birth anomalies in the same population simultaneously.

Twin studies offer a unique opportunity to investigate the potential roles of genetics and shared early exposures. Identical (monozygotic [MZ]) twins share 100% and fraternal (dizygotic [DZ]) twins share, on average, 50% of their genome. A comparison of disease concordance between MZ and DZ twin pairs can crudely estimate heritability.^9^^,^^10^ The excess of concordance for a disease among MZ twins comparing to DZ twins suggests a role for a genetic component in disease etiology.^11^ This is of increasing importance given that the birth rate for twins in the United States is increasing, with rates increasing from 18.9 per 1,000 live births in 1980 to 33.3 per 1,000 in 2009.^12^ However, there are limited epidemiological studies that investigate birth anomalies in twins; most were conducted in Europe and focused on genetic rather than environmental factors.^13^^–^^17^

Here we examine the prevalence of various birth anomalies, estimate the crude heritability, and investigate the association between selected birth characteristics and parental exposures and risk using data from the population-based California Twin Program (CTP).

## MATERIAL AND METHODS

### Study population

The CTP is a population-based cohort of twins born in California between 1908 and 1982. The development and representativeness of CTP have been described elsewhere.^18^^,^^19^ This study was based on information from a subset of twins born between 1957 and 1982, who were identified through birth records and California Department of Motor Vehicles (DMV) and returned and completed an updated 16-page questionnaire that included more detailed information on development and medical history, such as congenital conditions during the period of 1998 to 2001. A total of 28,050 individuals completed this questionnaire, representing 20,803 twin pairs, including both members of 7,247 pairs (double-respondent pairs), and one member of 13,556 pairs (single-respondent pairs). The crude overall response rate was 45.2%, which is comparable with or higher than similar cohort studies.^20^ In comparison with census data and California multiple birth records, the responding participants were representative of native California twins regarding to age, sex, zygosity, and residential distribution.^18^^,^^19^ The CTP was approved by the Institutional Review Board at the University of Southern California.

### Birth anomalies

The CTP questionnaire included a section on “Congenital Conditions”, listing 10 congenital conditions. In this study, we focused on eight anomalies: clubfoot, oral cleft, deafness, cerebral palsy, muscular dystrophy, spina bifida, strabismus (lazy eye), and congenital heart defects. Down syndrome and mental retardation were excluded from the analyses. Individuals were asked to report the presence of their own conditions (self-report) as well as those of their twins, brothers/sisters, and mothers/fathers (proxy-report). For double-respondent twin pairs, the disease condition was based on self-reports from both twins, whereas for single-respondent twin pairs, the disease condition was based on self- and proxy-report from the respondent twin. Concordant disease pairs were defined as those in which both twins had the same condition, and discordant disease pairs were those in which only one twin had the condition.

### Covariates

Participants’ characteristics (birth year, sex, and race/ethnicity) were obtained based on the self-reported questionnaires validated using birth records. Self-reported zygosity from the questionnaire was adjusted based on gender, similarity questions (“Were you as alike as two peas in a pod?”, “How frequent did good friends or close relatives get you mixed up?”) and confirmed by co-twins in double respondent-twin pairs, if available. Birth order was defined as the birth of the twin pair among all of their mother’s pregnancies that resulted in live births, and was categorized as 1^st^ birth, 2^nd^ birth, 3^rd^ birth, or 4^th^ or later birth. For the comparison within twin pairs, relative birthweight (“Which twin weighted more at birth?”) was coded as a binary variable: “1” for a twin member who indicated lower birth weight vs “0” for the co-twin with higher birth weight.

All parental exposures, including maternal age, mother’s and father’s smoking history, and mother’s and father’s education, were reported by the twins completing the questionnaire. Maternal age was grouped as 29 years or younger vs 30 years or older. Parent smoking history (not necessarily during the pregnancy) was summarized as neither parent smoked, only father smoked, only mother smoked, or both parent smoked. Parent’s (mother or father) education was treated as a dichotomous variable for each parent with 12 or less years’ education versus 13 or more years’ education.

In order to test the validity of the proxy responses from single-respondent twins’ questionnaires about their twins, agreement on shared factors (relative birth weight, birth order, maternal age, parental smoking, and parental education) was evaluated, comparing self-reports to proxy reports in double-respondent twin pairs. Proxy responses from single-respondent twins were included when agreement on the variable of interest was high (>70%). Responses from double-respondent twin pairs with consistent responses between members of the pair were included, but those with inconsistent responses were excluded from the analysis.

### Statistical analysis

All 20,803 twin pairs were considered in the analyses unless specified. The characteristics of study population were shown separately by affected twin pairs (twin pairs with at least one case of any selected birth anomaly) and unaffected twin pairs (twin pairs without any selected birth anomaly). For each birth anomaly, the frequency and percentage of concordant, discordant, and unaffected twin pairs were described by zygosity (MZ, DZ, or twins with unknown zygosity) and in total.

Pairwise concordance is the proportion of the pairs in which both twins are affected (concordant) among the pairs in which at least one twin is affected (concordant + discordant). The measure is used to predict the disease status of the co-twin given that one twin is affected. It was calculated as follows:

$$\begin{array}{ll} & \text{Pairwise}\;\text{concordance}\\ & \;=\text{No.}\;\text{concordant}\;\text{pairs}/(\text{No.}\;\text{concordant}\;\text{pairs}\\ & \;+\text{No.}\;\text{discordant}\;\text{pairs}) \end{array}$$

The standard error (SE) and χ^2^ test were calculated using methods developed by Witte et al.^21^ An excess in pairwise concordance among MZ compared to DZ twin pairs suggests that heritable factors contributed to the birth anomaly. Under random or complete twin ascertainment, such as the population-based CTP, pairwise concordance is unbiased and sufficient to estimate the disease concordance.^21^ In this analysis, only MZ (*n* = 6,752 pairs) and like-sex DZ (*n* = 7,326 pairs) pairs were included for fair comparisons, since factors other than inheritable factors (eg, hormone) in unlike-sex DZ pairs could contribute to the different outcomes.

In order to examine the effects of birth characteristics or parental exposures on each birth anomaly, two analytic approaches were used. First, a within-pair case-control study was performed in which the affected (case) and unaffected (control) twin’s characteristics were compared using conditional logistic regression. Since the twin pairs share all parental and demographic factors, no confounder was adjusted in the model. Second, for shared birth characteristics and parental exposures, a between-pair analysis was performed with the pair as the unit. Due to small numbers, birth anomaly discordant and concordant pairs were combined as “affected pairs” and were compared to unaffected control pairs using a logistic regression model. Based on prior knowledge and any significant association with each outcome from univariate tests, the between-pair analyses were adjusted for zygosity, gender, maternal age at the twins’ birth, parental education, and birth order when the adjusted variable was not tested as the main effect. Because there were too few affected pairs in the categories other than white for each anomaly, race/ethnicity was excluded from the analyses due to inadequate power. To further address the potential trend for the effect of parental smoking (from “neither parents smoked”, “only father smoked”, or “only mother smoked” to “both parents smoked”) and birth order (from 1^st^ birth, 2^nd^ birth, or 3^rd^ birth to 4^th^ birth or later) on the selected birth anomaly, trend tests with the corresponding contrasts were used to test for linear, quadratic, or cubic trends.

Sensitivity analyses were conducted by repeating the analyses among 7,247 double-respondent twin pairs, in order to examine the potential bias from the inclusion of single-respondent twin pairs in the main analyses.

All effects are reported as odds ratios (ORs) with 95% confidence intervals (CIs). If the cell number was less than 5, a Fisher’s exact test was used; otherwise, the likelihood ratio χ^2^ test was used. *P* values were reported for tests for concordant rates and association tests. Statistical analysis was performed using SAS software (Version 9.4; SAS institute, Inc., Cary, NC, USA).

## RESULTS

**Figure 1.**
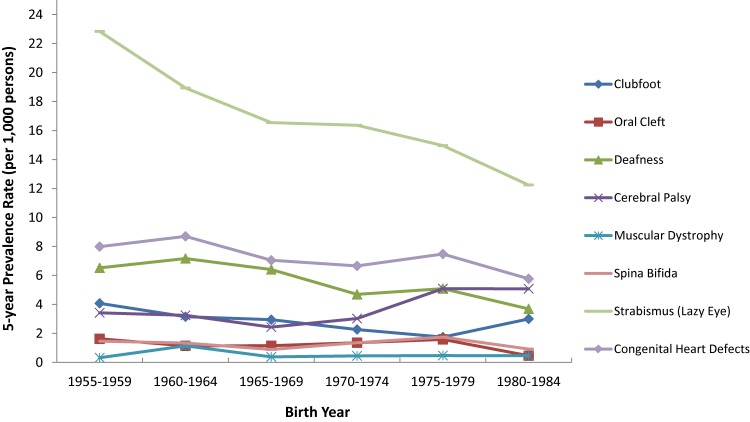
Five-year prevalence rate of selected birth anomalies (per 1,000 persons) in the California Twin Program (Birth cohort 1957–1982, *N* = 20,803 pairs)

The overall prevalence of any birth anomaly in California twins was 38 per 1,000 persons. From 1957 to 1982, when the study twins were born, there was a general declined trend for each selected anomaly, except for cerebral palsy, the rate of which decreased in the 1960s and then slightly increased in the 1980s (Figure 1). With the fluctuations across the investigated years, the largest overall drop rates were found in the prevalences of oral cleft (by 72%, from 1.63 to 0.46 per 1,000), strabismus (by 46%, from 22.8 to 12.2 per 1,000), deafness (by 43%, from 6.52 to 3.70 per 1,000), and spina bifida (by 37%, from 1.47 to 0.92 per 1,000). The decreasing trend was relatively mild for the other anomalies.

Table 1 showed the characteristics of California twin pairs in the study. Overall, females, DZ twins, and double-respondent twin pairs were more likely to report the presence of affected cases of a birth anomaly. The risk to have a birth anomaly in twins was higher in non-Hispanic whites than any other ethnic/racial group, which was consistent but not statistically significant for each selected anomaly. The affected twin pairs were more likely found in the families whose both parents smoked or whose father had lower education.

**Table 1.** Demographic characteristics by birth anomaly status among 20,803 twin pairs participating in the California Twin Program (Birth cohort 1957–1982).

		Affected twin pairs	Unaffected twin pairs
	
		*N* = 1,411	*N* = 19,392
**Sex pair**	Male-Male	359 (25.44)	5,456 (28.14)
	Male-Female	405 (28.7)	5,579 (28.77)
	Female-Female	647 (45.85)	8,357 (43.1)

**Zygosity**	MZ	414 (29.34)	6,338 (32.68)
	DZ	945 (66.97)	12,365 (63.76)
	Unknown^a^	52 (3.69)	689 (3.55)

**Race/ethnicity**	White	1,078 (76.4)	13,326 (68.72)
	Hispanic	142 (10.06)	2,888 (14.89)
	African American	42 (2.98)	908 (4.68)
	Others	102 (7.23)	1,565 (8.07)
	Unknown	47 (3.33)	705 (3.64)

**Birth order**	1st Birth	398 (28.21)	5,427 (27.99)
	2nd Birth	420 (29.77)	5,572 (28.73)
	3rd Birth	249 (17.65)	3,455 (17.82)
	4th or later Birth	298 (21.12)	4,347 (22.42)
	Unknown^a^	46 (3.26)	591 (3.05)

**Smoking**	Neither Smoked	351 (24.88)	5,822 (30.02)
	Only Father Smoked	241 (17.08)	3,881 (20.01)
	Only Mother Smoked	162 (11.48)	2,251 (11.61)
	Both smoked	543 (38.48)	6,018 (31.03)
	Unknown^a^	114 (8.08)	1,420 (7.32)

**Maternal age at birth**	<25	500 (35.44)	6,437 (33.19)
	25–29	392 (27.78)	5,340 (27.54)
	30–34	275 (19.49)	4,101 (21.15)
	≥35	149 (10.56)	2,188 (11.28)
	Unknown^a^	95 (6.73)	1,326 (6.84)

**Mother’s education**	12 or less years	646 (45.78)	8,871 (45.75)
	13 or more years	640 (45.36)	8,730 (45.02)
	Unknown^a^	125 (8.86)	1,791 (9.24)

**Father’s education**	12 or less years	587 (41.6)	7,613 (39.26)
	13 or more years	633 (44.86)	9,279 (47.85)
	Unknown^a^	191 (13.54)	2,500 (12.89)

**Response**	Double	540 (38.27)	6,707 (34.59)
	Single	871 (61.73)	12,685 (65.41)

**Mean age at completion of questionnaire (SD)**		31.83 (6.60)	30.96 (6.77)

Affected pairs are twin pairs with at least one selected birth anomaly; unaffected pairs are twin pairs without any selected birth anomaly.

^a^“Unknown” includes inconsistent measures within double-respondent twin pairs or missing values.

For each selected birth anomaly, there were consistently more concordant disease pairs among MZ than among DZ twins (Table 2). Although excluded from the main analyses, mental retardation was more likely to co-occur in individuals with an oral cleft, cerebral palsy, or Down syndrome (6.9%, 13.6%, and 16.3%, respectively; eTable 1). Strabismus was the most commonly reported anomaly in twins when the co-twin reported the presence of any other birth anomaly (7.7%–37.5%; eTable 2).

**Table 2.** Frequency of birth anomalies among 20,803 twin pairs by concordance and zygosity in the California Twin Program (Birth cohort 1957–1982)

Birth anomaly		Monozygotic twin pair	Dizygotic twin pair	Unknown zygosity	Total
**Clubfoot**	Concordant affected pairs	5 (0.07)	4 (0.03)	1 (0.13)	10 (0.05)
	Discordant affected pairs	17 (0.25)	82 (0.62)	1 (0.13)	100 (0.48)
	Unaffected pairs	6,730 (99.67)	13,224 (99.35)	739 (99.73)	20,693 (99.47)

**Oral cleft**	Concordant affected pairs	2 (0.03)	2 (0.02)	0 (0)	4 (0.02)
	Discordant affected pairs	7 (0.1)	36 (0.27)	1 (0.13)	44 (0.21)
	Unaffected pairs	6,743 (99.87)	13,272 (99.71)	740 (99.87)	20,755 (99.77)

**Deafness**	Concordant affected pairs	10 (0.15)	8 (0.06)	3 (0.4)	21 (0.1)
	Discordant affected pairs	70 (1.04)	128 (0.96)	4 (0.54)	202 (0.97)
	Unaffected pairs	6,672 (98.82)	13,174 (98.98)	734 (99.06)	20,580 (98.93)

**Cerebral palsy**	Concordant affected pairs	3 (0.04)	5 (0.04)	0 (0)	8 (0.04)
	Discordant affected pairs	44 (0.65)	81 (0.61)	7 (0.94)	132 (0.63)
	Unaffected pairs	6,705 (99.3)	13,224 (99.35)	734 (99.06)	20,663 (99.33)

**Muscular Dystrophy**	Concordant affected pairs	2 (0.03)	1 (0.01)	1 (0.13)	4 (0.02)
	Discordant affected pairs	3 (0.04)	12 (0.09)	2 (0.27)	17 (0.08)
	Unaffected pairs	6,747 (99.93)	13,297 (99.9)	738 (99.6)	20,782 (99.9)

**Spina bifida**	Concordant affected pairs	1 (0.01)	0 (0)	0 (0)	1 (0)
	Discordant affected pairs	16 (0.24)	33 (0.25)	3 (0.4)	52 (0.25)
	Unaffected pairs	6,735 (99.75)	13,277 (99.75)	738 (99.6)	20,750 (99.75)

**Strabismus**	Concordant affected pairs	33 (0.49)	27 (0.2)	5 (0.67)	65 (0.31)
	Discordant affected pairs	161 (2.38)	412 (3.1)	19 (2.56)	592 (2.85)
	Unaffected pairs	6,558 (97.13)	12,871 (96.7)	717 (96.76)	20,146 (96.84)

**Congenital heart defects**	Concordant affected pairs	13 (0.19)	14 (0.11)	2 (0.27)	29 (0.14)
	Discordant affected pairs	70 (1.04)	169 (1.27)	14 (1.89)	253 (1.22)
	Unaffected pairs	6,669 (98.77)	13,127 (98.63)	725 (97.84)	20,521 (98.64)

**Total**		**6,752**	**13,310**	**741**	**20,803**

In general, there was excess MZ compared to DZ concordance for each selected birth anomaly (Table 3). Of them, the most substantial MZ excess were found in clubfoot, oral cleft, and strabismus. Pairwise concordance of clubfoot in MZ and DZ like-sex pairs was 22.73% (SE, 8.93%) and 3.85% (SE, 2.67%), respectively. The relative risk of MZ compared to DZ like-sex pairs (concordance ratio) was 5.91 (*P* = 0.043). The concordance ratio for strabismus was 2.52 (*P* = 0.0001), given that 17.01% (SE, 2.70%) concordance in MZ versus 6.75% (SE, 1.58%) concordance in DZ like-sex. The concordance for oral cleft in MZ twins was 4.9 times that of DZ like-sex twins; however, the statistical test was insignificant (*P* = 0.224), probably due to the low prevalence. These findings were consistently reported using probandwise concordances or structural equation modelling methods (eTable 3).

**Table 3.** Pairwise Concordance Ratio between monozygotic twins (MZ, *N* = 6,752 pairs) and dizygotic like-sex twins (DZ like-sex, *N* = 7,326 pairs) for each birth anomaly in the California Twin Program (Birth cohort 1957–1982)

Proband	Zygosity	Concordant (*n*_11_)	Discordant (*n*_d_)	Pairwise concordance^a^ (%)	SE^b^ (%)	Concordance ratio^c^	*P*-value^d^
**Clubfoot**	MZ	5	17	22.73	8.93	5.91	0.043
	DZ like-sex	2	50	3.85	2.67		

**Oral cleft**	MZ	2	7	22.22	13.86	4.89	0.224
	DZ like-sex	1	21	4.55	4.44		

**Deafness**	MZ	10	70	12.50	3.70	2.25	0.129
	DZ like-sex	4	68	5.56	2.70		

**Cerebral palsy**	MZ	3	44	6.38	3.57	1.40	0.699
	DZ like-sex	2	42	4.55	3.14		

**Muscular dystrophy**	MZ	2	3	40.00	21.91	2.80	0.315
	DZ like-sex	1	6	14.29	13.23		

**Spina bifida**	MZ	1	16	5.88	5.71	Inf.	0.303
	DZ like-sex	0	18	0.00	0.00		

**Strabismus**	MZ	33	161	17.01	2.70	2.52	0.001
	DZ like-sex	17	235	6.75	1.58		

**Congenital heart defects**	MZ	13	70	15.66	3.99	1.90	0.121
	DZ like-sex	9	100	8.26	2.64		

^a^% Pairwise concordance = *n*_11_/(*n*_11_ + *n*_d_) × 100%.

^b^$SE=\sqrt{Var}=\sqrt{n_{11}n_{\text{d}}/{\left(n_{11}+n_{\text{d}}\right)}^{3}}$.

^c^Relative pairwise concordance = MZ (% pairwise concordance)/DZ like-sex (% pairwise concordance).

^d^Chi-square test to assess the difference in pairwise concordance rates between MZ and DZ like-sex.

Percent agreement among double-respondent pairs for shared factors ranged from 75% to 93% for shared factors (eTable 4), so proxy reports from single-respondent twins were included for all of these variables.

Parental smoking was associated with an increased risk of spina bifida (OR 3.48; 95% CI, 1.48–8.18) and strabismus (OR 1.61; 95% CI, 1.28–2.03; Table 4). All the other birth anomalies had non-significant increased risk associated with parental smoking. A significant quadratic trend of increasing risk was found when examining father smoked, mother smoked, or both parents smoked relative to non-smoking parents, for clubfoot, spina bifida, and strabismus (*P* = 0.029, 0.026, and 0.0005, respectively; Table 4). No linear or cubic trends were observed for any selected birth anomaly. For clubfoot and strabismus, the MZ:DZ concordance ratio among twins whose parents smoked was 5-fold and 3-fold higher, respectively, than that among twins with parents who were non-smokers (eTable 5). However, the MZ:DZ concordance for congenital heart defects was about 0.5 among twins whose parents smoked compared to those whose parents were non-smokers (eTable 5).

**Table 4.** Parental smoking status and risk of birth anomalies in the California Twin Program (Birth cohort 1957–1982, *N* = 20,803 pairs)

Affected (Concordant+Discordant) vs Unaffected		Neither Smoking *N* (%)	Only father smoking *N* (%)	Only mother smoking *N* (%)	Both smoking *N* (%)	Father only vs Neither		Mother only vs Neither		Both vs Neither		*P*-value for quadratic trend
		
						OR_Adj._^*^	95% CI	OR_Adj._^*^	95% CI	OR_Adj._^*^	95% CI	
**Clubfoot**	Affected (C+D) pairs	23 (0.45)	10 (0.30)	11 (0.60)	39 (0.76)	0.63	0.30–1.34	1.27	0.61–2.61	1.53	0.90–2.60	0.029
	Unaffected pairs	5,086 (99.55)	3,303 (99.70)	1,837 (99.40)	5,123 (99.24)	1 (ref)		1 (ref)		1 (ref)		

**Oral cleft**	Affected (C+D) pairs	13 (0.25)	6 (0.18)	7 (0.38)	10 (0.19)	0.67	0.25–1.80	1.47	0.58–3.72	0.72	0.31–1.67	0.517
	Unaffected pairs	5,096 (99.75)	3,307 (99.82)	1,841 (99.62)	5,152 (99.81)	1 (ref)		1 (ref)		1 (ref)		

**Deafness**	Affected (C+D) pairs	50 (0.98)	39 (1.18)	13 (0.70)	60 (1.16)	1.13	0.73–1.73	0.69	0.37–1.27	1.10	0.75–1.62	0.291
	Unaffected pairs	5,059 (99.02)	3,274 (98.82)	1,835 (99.30)	5,102 (98.84)	1 (ref)		1 (ref)		1 (ref)		

**Cerebral palsy**	Affected (C+D) pairs	36 (0.70)	19 (0.57)	10 (0.54)	37 (0.72)	0.92	0.52–1.62	0.83	0.41–1.68	1.16	0.72–1.85	0.923
	Unaffected pairs	5,073 (99.30)	3,294 (99.43)	1,838 (99.46)	5,125 (99.28)	1 (ref)		1 (ref)		1 (ref)		

**Muscular dystrophy**	Affected (C+D) pairs	6 (0.12)	1 (0.03)	1 (0.05)	5 (0.10)	0.23	0.03–1.90	0.42	0.05–3.54	0.70	0.21–2.36	0.864
	Unaffected pairs	5,103 (99.88)	3,312 (99.97)	1,847 (99.95)	5,157 (99.90)	1 (ref)		1 (ref)		1 (ref)		

**Spina bifida**	Affected (C+D) pairs	7 (0.14)	4 (0.12)	4 (0.22)	25 (0.48)	0.88	0.25–3.03	1.60	0.47–5.51	3.48	1.48–8.18	0.026
	Unaffected pairs	5,102 (99.86)	3,309 (99.88)	1,844 (99.78)	5,137 (99.52)	1 (ref)		1 (ref)		1 (ref)		

**Strabismus**	Affected (C+D) pairs	130 (2.54)	76 (2.29)	54 (2.92)	202 (3.91)	0.91	0.68–1.22	1.19	0.86–1.65	1.61	1.28–2.03	0.0005
	Unaffected pairs	4,979 (97.46)	3,237 (97.71)	1,794 (97.08)	4,960 (96.09)	1 (ref)		1 (ref)		1 (ref)		

**Congenital heart defects**	Affected (C+D) pairs	66 (1.29)	51 (1.54)	28 (1.52)	82 (1.59)	1.19	0.82–1.73	1.18	0.76–1.85	1.23	0.88–1.72	0.491
	Unaffected pairs	5,043 (98.71)	3,262 (98.46)	1,820 (98.48)	5,080 (98.41)	1 (ref)		1 (ref)		1 (ref)		

^*^Adjusted for zygosity, gender, maternal age, parental education and birth order.

Interestingly, maternal age (≥30 vs <30 years) was significantly associated with the decreasing risk of spina bifida (OR 0.29; 95% CI, 0.12–0.73) and congenital deafness (OR 0.68; 95% CI, 0.46–0.99) (eTable 6). No significant associations were found between maternal education and any selected birth anomaly. Paternal education was associated with a decreased risk of strabismus (OR 0.81; 95% CI, 0.66–0.99) and any birth anomaly (OR 0.87; 95% CI, 0.75–0.99) (eTable 7).

We also examined the effect of birth weight (within pairs) and birth order (between pairs) on the risk of birth anomalies. The occurrence of deafness (OR 1.63; 95% CI, 1.19–2.24), cerebral palsy (OR 1.83; 95% CI, 1.23–2.76), and congenital heart defects (OR 1.77; 95% CI, 1.34–2.35) were significantly associated with lower birth weight (eTable 8). Twin pairs who were first born compared to later born were more likely to be affected by strabismus (OR_2nd vs 1st_ 0.99; 95% CI, 0.79–1.24; OR_3rd vs 1st_ 0.76; 95% CI, 0.57–1.01; OR_≥4th vs 1st_ 0.75; 95% CI, 0.57–0.99; Quadratic trend *P* = 0.008; eTable 9).

## DISCUSSION

In this large population-based twin cohort, the overall prevalence of birth anomalies decreased slightly from 1957 to 1982. We observed strong evidence of an inherited susceptibility for various birth anomalies. The concordance for clubfoot in MZ twins was 5.9 times that of DZ like-sex twins, and that for strabismus was 2.5 times. In terms of parental exposures, parental smoking was associated with an increased risk of spina bifida and strabismus. Interestingly, advanced maternal age significantly decreased the risk of spina bifida and congenital deafness. A decreased risk of strabismus was found among twins who were not the first born in family or whose fathers had higher education. Comparing within twin pairs, twin member who had lower birth weight than the co-twin was more likely to have deafness, cerebral palsy, and congenital heart defects.

Previous studies have suggested an increased risk among twins for a number of different birth anomalies,^14^^–^^17^^,^^22^^–^^25^ although some population-based studies have found no excess risk among twins compared to singletons (eg, for oral cleft).^14^ In this twin study, the overall prevalence rates for congenital heart defects (7.5 per 1,000 live births) and oral cleft (1.25 per 1,000 live births) are comparable to the global rates in 2006 (cardiovascular system: 7.9 per 1,000 live births, oral cleft: 1.4 per 1,000 live births),^26^ indicating no excess risk of the two birth anomalies in California twins compared to the general population. However, the prevalence of clubfoot (2.9 per 1,000 live births) is close to that from the other twin study,^27^ about 2-fold higher than that of the general population.^4^ In addition, California twins appeared at a substantially greater risk for spina bifida (1.3 per 1,000 live births) comparing to the 2004–2006 general United States’ populations (0.35 per 1,000 live births).^1^ The large difference is probably due to the introduction of folate use during pregnancy in 1990s, which was after our twins were born. We found significant excess of MZ compared to DZ concordance for clubfoot and strabismus, suggesting an underlying genetic predisposition.^28^ Family-based studies and linkage studies have identified several candidate genes for clubfoot and strabismus, including *PITX1*^29^ and *STBMS1*,^30^^,^^31^ respectively. Moreover, the further different MZ:DZ concordance ratios comparing twins whose parents smoked to those with non-smoking parents provided potential evidence of gene-environment interactions for clubfoot, strabismus, and congenital heart defects. More research is needed to examine the molecular mechanisms.

Parental exposures, particularly maternal smoking, have been well-studied in relation to the risk of birth anomalies. Smoking may interfere with normal fetal development through a variety of mechanisms, including DNA damage, loss of essential nutrients, teratogenic effects, or fetal hypoxia.^32^ Consistent evidence is available for an effect of maternal smoking during pregnancy on an increased risk of oral cleft,^33^^,^^34^ clubfoot,^35^^–^^41^ strabismus,^31^^,^^42^^,^^43^ and congenital heart defects.^8^^,^^32^ A previous systematic review that included 17 studies found no association between maternal smoking and spina bifida.^8^ Paternal smoking during preconception has been linked to an increased risk of offspring anomalies, potentially as a result of sporadic DNA mutations in sperm or maternal secondhand smoke exposure.^44^ However, only borderline associations between paternal/secondhand smoking and clubfoot (OR 1.8; 95% CI, 0.97–3.37)^45^ and spina bifida (OR 1.9; 95% CI, 0.70–9.40)^46^ have been previously reported. In our study, we did not find any association between any birth anomaly and smoking status of either mother or father alone. However, when both parents smoked, the risks of spina bifida and strabismus in offspring were significantly increased. An quadratically increasing trend was also observed for the risk of clubfoot, spina bifida, and strabismus among offspring who had only father smoked, only mother smoked, and both parents smoked, suggesting that paternal smoking might have synergistic effect on the etiology of these three anomalies. One possible explanation for our failure to detect the significant association between maternal or paternal smoking alone and selected birth anomaly is potential misclassification of parental smoking measures from twins with no information of smoking level or time, such as during pregnancy. However, the agreement between offspring assessment of parental smoking status and parental self-report has been reported to be high (97%).^47^ Among our twins, who had limited prior knowledge of the relationship between birth anomaly and parental smoking at completion of questionnaires, more than 80% consistency was reported about which parent smoked, suggesting that any bias is likely to be non-differential, possibly leading to the attenuated effects.

It is well-documented that advanced maternal age increases birth prevalence of chromosomal abnormalities.^5^ Our study found decreased risk of spina bifida and congenital deafness among twins whose mothers were older than 29 years at delivery compared to younger mothers. There have been five studies that have investigated the associations between different types of birth anomalies and maternal age.^48^^–^^52^ One large population-based study in the Metropolitan Atlanta Congenital Defects Program (MACDP) involving in a total of 1,050,616 singleton infants found that young maternal age (14–19 years) was significantly associated with the elevated risk of anencephaly/spina bifida (OR 1.81; 95% CI, 1.30–2.52) and ear defects (OR 1.57, 95% CI, 1.10–1.49).^52^ Advanced maternal age was also associated with adverse pregnancy outcomes, such as low birth weight or infertility, possibly caused by the more frequent DNA mutations in germline cells and detrimental effects on decidual and placental development.^7^ On the other hand, advanced maternal age for first live birth correlated with higher income, better health care, higher socioeconomic status, and parental employment, which effects we may lack of power to test seperately.

This study was based on data collected from cross-sectional questionnaires and has several limitations and assumptions. First, sole reliance on self-reports can be a concern for this study. However, for a birth cohort before the 1980s, when no related registry was available, the conditions at birth could only be reported using self responses or the proxy (such as co-twin, or other relatives). Since the CTP questionnaire is a general survey designed decades ago with no specific study purpose and at a time when there was limited knowledge on birth anomalies, recall bias is likely to be limited. More than 75% agreements on exposures of interest in this study within our twin pairs suggest that the measures from self-report or proxy-report when self response was missing are reasonably valid and reliable. In addition, self-reported zygosity has shown more than 95% consistency with genetically determined zygosity in several studies, including a study with 600 pairs of the CTP twins.^19^^,^^53^^,^^54^ All together, the misclassifications from self-reports are more likely to be non-differential, leading the results towards the null. And our major findings have been repeated in analyses conducted only using consistent measures within double-respondent twin pairs. Second, potential selection bias may arise from DMV linkage for twin identification in the CTP. Some conditions in birth anomalies, such as physical disability or intellectual delays, could affect the capability to be in the DMV records or complete the questionnaires, thus resulting in less likelihood of being included in the study and a subsequently lower prevalence or attenuation of the concordance ratios, since the concordant affected twin pairs usually have more severe conditions than the discordant affected pairs. As a result, Down syndrome, the most affected anomaly was excluded from this study, despite the popular interest in this condition. Third, in a classic twin study, MZ and DZ twins are assumed to share unmeasured environmental factors equally (“Equal Environment assumption”), so excess concordance among MZ over DZ twins suggests heritable influences. However, any violation of this assumption, for example, a different uterine environment in DZ comparing to MZ twins, could result in an exaggerated genetic contribution to the selected birth anomaly observed in this study. Lastly, because twins are uncommon and most of birth anomalies have low prevalence in the general population, this study suffers an inadequate power to separate the concordant and discordant affected twin pairs in association studies or test other interesting factors (eg ethnicity or birth places), which also leads to difficulty in investigating gene-environment interactions using more sophiscated twin models, although the CTP is a large population-based cohort.

Using twins as the study population to investigate birth anomaly has the unique strengths over a traditional observational study. The major advantage of a twin study is to disentangle the genetic factors, shared and unshared environmental factors for a disease.^55^^,^^56^^,^^57^ In addition, the self-reported information in twins can be validated by the co-twin, providing more confidence than a standard study of unrelated persons. Finally, the most current knowledge of birth anomalies in twins were from published studies in the Europe, where the population is relatively homogeneous and very different from the United States’ population. This study is the first twin study in the United States to explore various types of birth anomalies, to examine the heritable contributions, as well as to provide evidence of their associations with a set of birth characteristics and parental exposures. The unique twin study design may provide valuable clues to further understand the etiology, thus directing future studies.

## APPENDIX A. SUPPLEMENTARY DATA

eTable 1. Co-occurrence of multiple birth anomalies in the same individual from the California Twin Program (Birth cohort 1957–1982, N = 20,803 pairs)

eTable 2. Co-occurrence of birth anomalies in twin pairs from the California Twin Program (Birth cohort 1957–1982, N = 20,803 pairs)

eTable 3. Comparisons of heritability estimation for each selected birth anomaly using concordance methods and structural equation modelling method in the California Twin Program

eTable 4. Percentage agreement for the shared birth-related factors or parental exposures within double-respondent twin pairs (N = 7,247 pairs) from the California Twin Program (Birth cohort 1957–1982)

eTable 5. Pairwise Concordance Ratio between monozygotic twins (MZ, N = 6,752 pairs) and dizygotic like-sex twins (DZ like-sex, N = 7,326 pairs) for each birth anomaly identified in California Twin Program (Birth cohort 1957–1982), stratified by parental smoking status

eTable 6. Maternal age and risk of birth anomalies in the California Twin Program (Birth cohort 1957–1982, N = 20,803 pairs)

eTable 7. Parental education and risk of birth anomalies in the California Twin Program (Birth cohort 1957–1982, N = 20,803 pairs)

eTable 8. Low birth weight and risk of birth anomalies in the California Twin Program (Birth cohort 1957–1982) in total or stratified by MZ and DZ like-sex twin pairs

eTable 9. Birth order and risk of birth anomalies in the California Twin Program (Birth cohort 1957–1982, N = 20,803 pairs)
