# Supplementary material for: Birth Anomalies in Monozygotic and Dizygotic Twins: Results From the California Twin Registry
Source: J Epidemiol. 2019 Jan 5;29(1):18–25. doi: 10.2188/jea.JE20170159 (PMC6290277; doi:10.2188/jea.JE20170159)
Supplement: Supplementary file 1 [file je-29-018-s001.pdf]

eTable 1. Co-occurrence of multiple birth anomalies in the same individual from the California Twin Program (Birth cohort 1957─1982, N=20,803 pairs)

Proband disease (column)	Clubfoot	Oral Cleft	Deafness	Cerebral Palsy	Muscular Dystrophy	Down Syndrome[a]	Mental Retardatio[a]	Spina Bifida	Strabismus (Lazy Eye)	Congenital Heart Defects	Other[a]
**Clubfoot**	120 (83.92)	2 (2.78)	1 (0.35)	2 (0.91)	2 (4.65)	0 (0)	2 (0.9)	1 (1.39)	5 (0.62)	4 (1.13)	4 (1.31)
**Oral Cleft**	2 (1.4)	52 (72.22)	1 (0.35)	1 (0.45)	2 (4.65)	1 (2.33)	5 (2.24)	0 (0)	3 (0.37)	2 (0.56)	3 (0.98)
**Deafness**	1 (0.7)	1 (1.39)	244 (86.22)	5 (2.27)	2 (4.65)	2 (4.65)	5 (2.24)	1 (1.39)	9 (1.11)	8 (2.25)	5 (1.63)
**Cerebral Palsy**	2 (1.4)	1 (1.39)	5 (1.77)	148 (67.27)	3 (6.98)	0 (0)	30 (13.45)	3 (4.17)	18 (2.22)	7 (1.97)	3 (0.98)
**Muscular Dystrophy**	2 (1.4)	2 (2.78)	2 (0.71)	3 (1.36)	25 (58.14)	1 (2.33)	2 (0.9)	2 (2.78)	1 (0.12)	2 (0.56)	1 (0.33)
**Down Syndrome[a]**	0 (0)	1 (1.39)	2 (0.71)	0 (0)	1 (2.33)	27 (62.79)	7 (3.14)	1 (1.39)	0 (0)	4 (1.13)	0 (0)
**Mental Retardation[a]**	2 (1.4)	5 (6.94)	5 (1.77)	30 (13.64)	2 (4.65)	7 (16.28)	143 (64.13)	4 (5.56)	11 (1.36)	7 (1.97)	7 (2.29)
**Spina Bifida**	1 (0.7)	0 (0)	1 (0.35)	3 (1.36)	2 (4.65)	1 (2.33)	4 (1.79)	54 (75)	3 (0.37)	2 (0.56)	1 (0.33)
**Strabismus (Lazy Eye)**	5 (3.5)	3 (4.17)	9 (3.18)	18 (8.18)	1 (2.33)	0 (0)	11 (4.93)	3 (4.17)	722 (89.25)	20 (5.63)	17 (5.56)
**Congenital Heart Defects**	4 (2.8)	2 (2.78)	8 (2.83)	7 (3.18)	2 (4.65)	4 (9.3)	7 (3.14)	2 (2.78)	20 (2.47)	286 (80.56)	13 (4.25)
**Other[a]**	4 (2.8)	3 (4.17)	5 (1.77)	3 (1.36)	1 (2.33)	0 (0)	7 (3.14)	1 (1.39)	17 (2.1)	13 (3.66)	252 (82.35)
**Total**	143	72	283	220	43	43	223	72	809	355	306

Values are shown as n (%).[a] Conditions that were not included in the main analyses

eTable 2. Co-occurrence of birth anomalies in twin pairs from the California Twin Program (Birth cohort 1957–1982, N=20,803 pairs)

Proband disease (Column) Co-twin's disease (Row)	Clubfoot	Oral Cleft	Deafness	Cerebral Palsy	Muscular Dystrophy	Spina Bifida	Strabismus (Lazy Eye)	Congenital Heart Defects
**Clubfoot**	10 (41.67)	2 (15.38)	0 (0)	2 (8.33)	0 (0)	1 (12.5)	3 (3.19)	6 (11.54)
**Oral Cleft**	2 (8.33)	4 (30.77)	2 (4.88)	1 (4.17)	2 (20)	1 (12.5)	1 (1.06)	0 (0)
**Deafness**	0 (0)	2 (15.38)	21 (51.22)	4 (16.67)	1 (10)	0 (0)	7 (7.45)	5 (9.62)
**Cerebral Palsy**	2 (8.33)	1 (7.69)	4 (9.76)	8 (33.33)	1 (10)	1 (12.5)	4 (4.26)	2 (3.85)
**Muscular Dystrophy**	0 (0)	2 (15.38)	1 (2.44)	1 (4.17)	4 (40)	1 (12.5)	1 (1.06)	0 (0)
**Spina Bifida**	1 (4.17)	1 (7.69)	0 (0)	1 (4.17)	1 (10)	1 (12.5)	3 (3.19)	0 (0)
**Strabismus (Lazy Eye)**	3 (12.5)	1 (7.69)	7 (17.07)	4 (16.67)	1 (10)	3 (37.5)	65 (69.15)	10 (19.23)
**Congenital Heart Defects**	6 (25)	0 (0)	5 (12.2)	2 (8.33)	0 (0)	0 (0)	10 (10.64)	29 (55.77)
**Total**	24	13	41	24	10	8	94	52

Values are shown as n (%).

eTable 3. Comparisons of heritability estimation for each selected birth anomaly using concordance methods and structural equation modelling method in the California Twin Program

Birth Anomaly	Pairwise Concordance Ratio[a] (Table 3)	Probandwise Concordance Ratio[b]	SEM estimates[c]	
			Probandwise Concordance Ratio	Heritability (SE)
**Clubfoot**	5.91	5.00	5.44	0.80 (0.21)
**Oral Cleft**	4.89	4.18	4.50	0.65 (0.28)
**Deafness**	2.25	2.11	2.00	0.36 (0.18)
**Cerebral Palsy**	1.40	1.38	1.00	0.00 (0.00)
**Muscular Dystrophy**	2.80	2.29	2.70	0.40 (0.33)
**Spina Bifida**	Inf.	Inf.	8.00	0.55 (0.18)
**Strabismus**	2.52	2.30	2.89	0.58 (0.12)
**Congenital Heart Defects**	1.90	1.78	2.30	0.38 (0.17)

SE, standard error; SEM, structural equation modeling.

[a] Pairwise concordance ratios were computed as shown in Table 3 by comparing pairwise concordance between 6,752 monozygotic (MZ) twin pair and 7,326 dizygotic (DZ) like-sex twin pairs, where pairwise concordance was calculated for MZ or DZ-like sex twin pairs as $n_{11}/(n_{11}+n_d)$, and n_{11} is number of concordant twin pairs, n_d is number discordanct twin pairs.

[b] Probandwise concordance ratios were computed from Table 3 by comparing probandrwise concordance between 6,752 MZ twin pair and 7,326 DZ like-sex twin pairs, where probandwise concordance was calculated for MZ or DZ-like sex twin pairs as $2n_{11}/(2n_{11}+n_d)$, and n_{11} is number of concordant twin pairs, n_d is number of discordanct twin pairs.

[c] Structual equation modelling (SEM) estimates were computed using R package "mets" by comparing 6,752 MZ and 13,310 DZ twin pairs after adjustment for gender. The estimates included probandwise concordace ratio for MZ vs. DZ as well as the heritability estimates based on a classic ACE twin model. In a ACE model, "A" stands for additive genetic component, while "C" and "E" are shared and non-shared environmental components, respectively, in which heritability was calculated with Var(A)/(Var(A)+Var(C)+Var(E)) as the contribution of additive genetic factors to the total variance.

eTable 4. Percentage agreement for the shared birth-related factors or parental exposures within double-respondent twin pairs (N=7,247 pairs) from the California Twin Program (Birth cohort 1957–1982)

Agreement on the shared factors within twin pairs	%
Did your parents smoke cigarettes? (Neither parents vs. Father only vs. Mother only vs. Both parents)	81.1
How old was your biological mother when you were born? (<30 vs. ≥30 years old)	91.8
How many years of school did your mother finish? (≤12 vs. >12 years)	81.3
How many years of school did your father finish? (≤12 vs. >12 years)	80.3
Which twin weighed more at birth? (You vs. Your twin)	75.0
Considering all of your mother's pregnancies that resulted in live births, which pregnancy resulted in the birth of you and your twin? (1st vs. 2nd vs. 3rd. vs 4th or later).	92.7

eTable 5. Pairwise Concordance Ratio between monozygotic twins (MZ, N=6,752 pairs) and dizygotic like-sex twins (DZ like-sex, N=7,326 pairs) for each birth anomaly identified in California Twin Program (Birth cohort 1957–1982), stratified by parental smoking status

Parent Smoking history		At least one parent smoked			Neither parent smoked			CRR[a]
**Proband**	**Zygosity**	**Pairwise concordance (%)**	**Concordance Ratio**	**P-value**	**Pairwise concordance (%)**	**Concordance Ratio**	**P-value**	
**Clubfoot**	MZ	25.00	10.00	0.020	16.67	2.00	1.000	5.00
	DZ like-sex	2.50			8.33			
**Oral Cleft**	MZ	33.33	5.67	0.155	0.00	N.A	N.A	N.A
	DZ like-sex	5.88			0.00			
**Deafness**	MZ	12.77	1.91	0.329	11.11	Inf.	0.539	N.A
	DZ like-sex	6.67			0.00			
**Cerebral Palsy**	MZ	3.33	0.47	0.605	11.76	Inf.	0.516	N.A
	DZ like-sex	7.14			0.00			
**Muscular Dystrophy**	MZ	50.00	N.A	0.167	0.00	0.00	1.000	N.A
	DZ like-sex	0.00			50.00			
**Spina Bifida**	MZ	10.00	Inf.	0.400	0.00	N.A	N.A	N.A
	DZ like-sex	0.00			0.00			
**Strabismus (Lazy Eye)**	MZ	19.66	3.40	0.0003	13.11	1.16	1.000	2.93
	DZ like-sex	5.79			11.32			
**Congenital Heart Defects**	MZ	16.33	1.73	0.274	16.67	3.67	0.226	0.47
	DZ like-sex	9.41			4.55			

[a] CRR, Concordance Rate Ratio comparing parent smoking vs. neither parent smoking

eTable 6. Maternal age and risk of birth anomalies in the California Twin Program (Birth cohort 1957–1982, N=20,803 pairs)

**Affected (Concordant+Discordant) vs. Unaffected**		**<30 N (%)**	**≥30 N (%)**	**≥30 vs. <30**		
				**OR_{Adj}[a]**	**95% CI**	**P-value**
**Clubfoot**	**Affected (C+D) pairs**	63 (0.58)	23 (0.41)	0.73	0.43–1.24	0.246
	**Unaffected pairs**	10,725 (99.42)	5,639 (99.59)	1 (ref)		
**Oral Cleft**	**Affected (C+D) pairs**	21 (0.19)	16 (0.28)	1.55	0.75–3.20	0.240
	**Unaffected pairs**	10,767 (99.81)	5,646 (99.72)	1 (ref)		
**Deafness**	**Affected (C+D) pairs**	122 (1.13)	47 (0.83)	0.68	0.47–0.99	0.045
	**Unaffected pairs**	10,666 (98.87)	5,615 (99.17)	1 (ref)		
**Cerebral Palsy**	**Affected (C+D) pairs**	81 (0.75)	30 (0.53)	0.69	0.43–1.09	0.112
	**Unaffected pairs**	10,707 (99.25)	5,632 (99.47)	1 (ref)		
**Muscular Dystrophy**	**Affected (C+D) pairs**	13 (0.12)	1 (0.02)	0.18	0.02–1.52	0.116
	**Unaffected pairs**	10,775 (99.88)	5,661 (99.98)	1 (ref)		
**Spina Bifida**	**Affected (C+D) pairs**	37 (0.34)	6 (0.11)	0.29	0.12–0.73	0.008
	**Unaffected pairs**	10,751 (99.66)	5,656 (99.89)	1 (ref)		
**Strabismus (Lazy Eye)**	**Affected (C+D) pairs**	325 (3.01)	184 (3.25)	1.20	0.98–1.47	0.084
	**Unaffected pairs**	10,463 (96.99)	5,478 (96.75)	1 (ref)		
**Congenital Heart Defects**	**Affected (C+D) pairs**	165 (1.53)	79 (1.40)	0.84	0.62–1.13	0.243
	**Unaffected pairs**	10,623 (98.47)	5,583 (98.60)	1 (ref)		

CI, confidence interval; OR, odds ratio.
[a] Adjusted for zygosity, gender, parental education and birth order

eTable 7. Parental education and risk of birth anomalies in the California Twin Program (Birth cohort 1957–1982, N=20,803 pairs)

Affected (Concordant+Discordant) vs. Unaffected		Mother's Education					Father's Education				
		≤12 N (%)	>12 N (%)	>12 vs. ≤12			≤12 N (%)	>12 N (%)	>12 vs. ≤12		
				OR_{Adj}[a]	95% CI	P-value			OR_{Adj}[a]	95% CI	P-value
Clubfoot	Affected (C+D) pairs	52 (0.64)	34 (0.41)	0.61	0.37–1.00	0.051	43 (0.58)	43 (0.48)	1.04	0.64–1.69	0.879
	Unaffected pairs	8,083 (99.36)	8,281 (99.59)	1 (ref)			7,395 (99.42)	8,969 (99.52)	1 (ref)		
Oral Cleft	Affected (C+D) pairs	18 (0.22)	19 (0.23)	1.10	0.51–2.34	0.810	18 (0.24)	19 (0.21)	0.79	0.37–1.68	0.539
	Unaffected pairs	8,117 (99.78)	8,296 (99.77)	1 (ref)			7,420 (99.76)	8,993 (99.79)	1 (ref)		
Deafness	Affected (C+D) pairs	97 (1.19)	72 (0.87)	0.83	0.58–1.19	0.311	90 (1.21)	79 (0.88)	0.82	0.58–1.17	0.272
	Unaffected pairs	8,038 (98.81)	8,243 (99.13)	1 (ref)			7,348 (98.79)	8,933 (99.12)	1 (ref)		
Cerebral Palsy	Affected (C+D) pairs	42 (0.52)	69 (0.83)	1.55	0.99–2.43	0.053	42 (0.56)	69 (0.77)	1.10	0.71–1.72	0.668
	Unaffected pairs	8,093 (99.48)	8,246 (99.17)	1 (ref)			7,396 (99.44)	8,943 (99.23)	1 (ref)		
Muscular Dystrophy	Affected (C+D) pairs	9 (0.11)	5 (0.06)	0.70	0.20–2.42	0.568	9 (0.12)	5 (0.06)	0.52	0.15–1.80	0.302
	Unaffected pairs	8,126 (99.89)	8,310 (99.94)	1 (ref)			7,429 (99.88)	9,007 (99.94)	1 (ref)		
Spina Bifida	Affected (C+D) pairs	21 (0.26)	22 (0.26)	1.17	0.58–2.33	0.665	21 (0.28)	22 (0.24)	0.84	0.42–1.68	0.627
	Unaffected pairs	8,114 (99.74)	8,293 (99.74)	1 (ref)			7,417 (99.72)	8,990 (99.76)	1 (ref)		
Strabismus (Lazy Eye)	Affected (C+D) pairs	231 (2.84)	278 (3.34)	1.26	0.98–1.55	0.051	237 (3.19)	272 (3.02)	0.81	0.66–0.998	0.047
	Unaffected pairs	7,904 (97.16)	8,037 (96.66)	1 (ref)			7,201 (96.81)	8,740 (96.98)	1 (ref)		
Congenital Heart Defects	Affected (C+D) pairs	121 (1.49)	123 (1.48)	1.05	0.78–1.41	0.753	113 (1.52)	131 (1.45)	0.97	0.72–1.30	0.833
	Unaffected pairs	8,014 (98.51)	8,192 (98.52)	1 (ref)			7,325 (98.48)	8,881 (98.55)	1 (ref)		

CI, confidence interval; OR, odds ratio.
[a] Adjusted for zygosity, gender, maternal age and birth order

eTable 8. Low birth weight and risk of birth anomalies in the California Twin Program (Birth cohort 1957–1982) in total or stratified by MZ and DZ like-sex twin pairs

Discordant Pairs	Total (N=20,803)					MZ (N=6,752)					DZ like-sex (N=7,326)				
	b/c[a]	OR_{Unadj}	95% CI		P-Value	b/c[a]	OR_{Unadj}	95% CI		P-Value	b/c[a]	OR_{Unadj}	95% CI		P-Value
			Low	High				Low	High				Low	High	
**Clubfoot**	48/44	1.09	0.71	1.68	0.755	7/8	0.88	0.27	2.76	1.000	24/24	1.00	0.54	1.84	1.000
**Oral Cleft**	20/17	1.18	0.59	2.39	0.743	3/3	1.00	0.13	7.47	1.000	10/7	1.43	0.49	4.42	0.629
**Deafness**	109/67	1.63	1.19	2.24	0.002	32/28	1.14	0.67	1.97	0.699	39/23	1.70	0.99	2.97	0.056
**Cerebral Palsy**	73/40	1.83	1.23	2.76	0.003	24/12	2.00	0.96	4.39	0.065	21/17	1.24	0.62	2.49	0.627
**Muscular Dystrophy**	5/7	0.71	0.18	2.61	0.774	1/1	1.00	0.01	78.50	1.000	0/4	0.19	<0.001	1.12	0.125
**Spina Bifida**	26/17	1.53	0.80	3.00	0.222	9/4	2.25	0.63	10.00	0.267	8/9	0.89	0.30	2.60	1.000
**Strabismus (Lazy Eye)**	264/257	1.03	0.86	1.23	0.793	69/75	0.92	0.65	1.29	0.677	102/104	0.98	0.74	1.30	0.945
**Congenital Heart Defects**	143/81	1.77	1.34	2.35	<0.0001	36/25	1.44	0.84	2.50	0.200	60/30	2.00	1.27	3.21	0.002

CI, confidence interval; DZ, dizygotic; OR, odds ratio; MZ, monozygotic.
[a] Matched 2x2 table cell number b and c: b=Exposed case & Unexposed co-twin, c=Unexposed case & Exposed co-twin. Here, the “exposed” twin is the twin member with lower birth weight in a twin pair compared to their co-twin (“unexposed”) based on their self-reports.

eTable 9. Birth order and risk of birth anomalies in the California Twin Program (Birth cohort 1957–1982, N=20,803 pairs)

Affected (Concordant+Discordant) vs. Unaffected		1st Birth N (%)	2nd Birth N (%)	3rd Birth N (%)	4th or later Birth N (%)	2nd vs. 1st		3rd vs. 1st		4th or later vs. 1st		P-value for quadratic trend
						OR_{Adj}[a]	95% CI	OR_{Adj}[a]	95% CI	OR_{Adj}[a]	95% CI	
**Clubfoot**	Affected (C+D) pairs	30 (0.61)	26 (0.52)	14 (0.46)	16 (0.45)	0.84	0.50–1.43	0.74	0.38–1.41	0.70	0.36–1.38	0.323
	Unaffected pairs	4,864 (99.39)	4,946 (99.48)	3,000 (99.54)	3,554 (99.55)	1 (ref)		1 (ref)		1 (ref)		
**Oral Cleft**	Affected (C+D) pairs	10 (0.20)	14 (0.28)	4 (0.13)	9 (0.25)	1.29	0.57–2.92	0.54	0.16–1.76	0.89	0.32–2.44	0.211
	Unaffected pairs	4,884 (99.80)	4,958 (99.72)	3,010 (99.87)	3,561 (99.75)	1 (ref)		1 (ref)		1 (ref)		
**Deafness**	Affected (C+D) pairs	41 (0.84)	59 (1.19)	29 (0.96)	40 (1.12)	1.45	0.97–2.16	1.21	0.75–1.98	1.53	0.94–2.48	0.474
	Unaffected pairs	4,853 (99.16)	4,913 (98.81)	2,985 (99.04)	3,530 (98.88)	1 (ref)		1 (ref)		1 (ref)		
**Cerebral Palsy**	Affected (C+D) pairs	40 (0.82)	29 (0.58)	22 (0.73)	20 (0.56)	0.75	0.46–1.22	1.05	0.61–1.79	0.94	0.52–1.71	0.532
	Unaffected pairs	4,854 (99.18)	4,943 (99.42)	2,992 (99.27)	3,550 (99.44)	1 (ref)		1 (ref)		1 (ref)		
**Muscular Dystrophy**	Affected (C+D) pairs	7 (0.14)	2 (0.04)	4 (0.13)	1 (0.03)	0.29	0.06–1.42	1.11	0.32–3.88	0.31	0.04–2.72	0.921
	Unaffected pairs	4,887 (99.86)	4,970 (99.96)	3,010 (99.87)	3,569 (99.97)	1 (ref)		1 (ref)		1 (ref)		
**Spina Bifida**	Affected (C+D) pairs	18 (0.37)	6 (0.12)	13 (0.43)	6 (0.17)	0.36	0.14–0.90	1.50	0.72–3.12	0.78	0.29–2.11	0.099
	Unaffected pairs	4,876 (99.63)	4,966 (99.88)	3,001 (99.57)	3,564 (99.83)	1 (ref)		1 (ref)		1 (ref)		
**Strabismus (Lazy Eye)**	Affected (C+D) pairs	163 (3.33)	167 (3.36)	80 (2.65)	99 (2.77)	0.99	0.79–1.24	0.76	0.57–1.01	0.75	0.57–0.998	0.0081
	Unaffected pairs	4,731 (96.67)	4,805 (96.64)	2,934 (97.35)	3,471 (97.23)	1 (ref)		1 (ref)		1 (ref)		
**Congenital Heart defects**	Affected (C+D) pairs	74 (1.51)	63 (1.27)	48 (1.59)	59 (1.65)	0.86	0.61–1.20	1.13	0.78–1.65	1.23	0.83–1.81	0.093
	Unaffected pairs	4,820 (98.49)	4,909 (98.73)	2,966 (98.41)	3,511 (98.35)	1 (ref)		1 (ref)		1 (ref)		

CI, confidence interval; OR, odds ratio.
[a] Adjusted for zygosity, gender, maternal age and parental education.
